# Tumor-associated neutrophils in pancreatic ductal adenocarcinoma: mechanisms and therapeutic targeting

**DOI:** 10.3389/fimmu.2026.1806714

**Published:** 2026-04-07

**Authors:** Xiaoyong Wu, Weixiong Zhu, Weili Chen, Liqin Ruan

**Affiliations:** 1 Department of Hepatobiliary Surgery, Jiujiang City Key Laboratory of Cell Therapy, Jiujiang No. 1 People’s Hospital, Jiujiang, Jiangxi, China; 2 The Second Clinical Medical College, Lanzhou University, Lanzhou, China; 3 Department of General Surgery, The Second Hospital of Lanzhou University, Lanzhou, China

**Keywords:** immunosuppression, neutrophil extracellular traps, pancreatic ductal adenocarcinoma, tumor microenvironment, tumor-associated neutrophils

## Abstract

Pancreatic ductal adenocarcinoma (PDAC) remains one of the most lethal malignancies, characterized by profound therapeutic resistance and a 5-year survival rate below 10%. This prognosis is largely driven by a highly immunosuppressive tumor microenvironment (TME), in which tumor-associated neutrophils (TANs) serve as pivotal regulators. Upon recruitment to the TME, neutrophils undergo functional polarization into distinct phenotypes that either antagonize or facilitate tumor progression. This review synthesizes recent advances in PDAC research, delineating the ontogeny, subpopulation heterogeneity, and molecular mechanisms governing the pro- or anti-tumorigenic effects of TANs. We emphasize the regulatory crosstalk between TANs and the immune microenvironment, highlighting key signaling axes such as TGF-β and C-X-C chemokine receptor 2 (CXCR2) pathways. Furthermore, we evaluate TAN-targeted therapeutic strategies, categorizing them into recruitment inhibition, functional reprogramming, and immunosuppression disruption. Finally, we discuss translational challenges, including biomarker development and the shift from neutrophil depletion to functional reprogramming, offering perspectives for overcoming therapeutic resistance in PDAC.

## 1 Introduction

PDAC remains a formidable clinical challenge, characterized by insidious onset, early systemic dissemination, and profound therapeutic resistance (1). Despite the potential for curative surgical resection, the majority of patients present with unresectable disease at diagnosis, and those who undergo surgery face a high risk of postoperative recurrence (2). PDAC has shown dismal responses to conventional chemotherapy, radiotherapy, and immune checkpoint blockers (ICBs). The recalcitrance is primarily attributed to its unique “cold” TME (3, 4).

The TME is characterized by a dense extracellular scaffold populated by activated fibroblastic lineages (e.g., PSCs), immune infiltrates, and a suppressive milieu of secretory signaling molecules (5). Among the infiltrating immune cells, neutrophils emerge as pivotal players and are closely correlated with dismal clinical outcomes (6, 7). Within the TME, TANs acquire distinct functional phenotypes and, in turn, reciprocally remodel the TME (8). They modulate tumor biological behavior through multifaceted mechanisms, including the release of proteases and cytokines, the formation of neutrophil extracellular traps (NETs), and the orchestration of an immunosuppressive landscape (9). Although their pro-tumorigenic roles are well-documented, neutrophils are not a functionally monolithic population (10). Recent research has unveiled a high degree of TAN heterogeneity, delineating multiple functionally distinct subpopulations within the PDAC microenvironment (11). Beyond this structural diversity, TANs demonstrate remarkable functional plasticity, capable of transitioning between pro-tumorigenic and anti-tumorigenic states in response to microenvironmental cues (12, 13). This plasticity, coupled with their heterogeneity, positions TANs as active and potentially targetable nodes for therapeutic intervention.

In this review, we elucidate the molecular mechanisms governing TAN-mediated tumor modulation, analyze their contributions to microenvironmental remodeling and functional plasticity, and evaluate their prognostic significance. Furthermore, we summarize emerging TAN-targeted therapeutic strategies, providing insights into their potential for overcoming the current limitations in PDAC treatment.

## 2 Recruitment and polarization of TANs

### 2.1 Recruitment of TANs

In PDAC, a complex interplay between tumor cells, stromal constituents, and resident immune populations mediates the recruitment of circulating neutrophils into the TME (14). Driven by an array of chemokines and cytokines, these cells are subsequently converted into TANs, characterized by extended longevity and augmented immunosuppressive potency relative to circulating neutrophils (15).

CXCR2, a G protein-coupled receptor (GPCR) primarily expressed on myeloid-derived cells, specifically recognizes human C-X-C motif chemokines (CXCLs), including C-X-C motif chemokine ligand 1/2/3/5/6/7/8 (CXCL1/2/3/5/6/7/8), secreted within the tumor stroma (16). The CXCR2 signaling axis and its cognate ligands represent the core recruitment route for TANs (17, 18). In PDAC, KRAS and TP53 represent the most frequently mutated genes (19–21). Constitutive activation of the KRAS pathway induces the secretion of G-CSF, thereby driving the infiltration of TANs into the TME (22). Concurrently, TP53 mutations exacerbate this process through multiple mechanisms: the loss of the tumor suppressor EHF (ESE3) derepresses CXCL1 transcription (23), while gain-of-function mutant p53 (e.g., p53R172H) directly upregulates CXCL2 and CXCL5. Interestingly, recruited neutrophils themselves can secrete CXCL2, establishing an autocrine and paracrine positive feedback loop that sustains and amplifies the recruitment signal. Beyond genetic drivers, epigenetic and inflammatory signaling further refine the recruitment landscape (24). Loss of the lysine demethylase KDM6A or the histone H3K36 trimethyltransferase SETD2 significantly elevates the production of CXCL1 and GM-CSF, with KDM6A deficiency notably promoting both recruitment and the subsequent formation of neutrophil extracellular traps (NETs) (25, 26). The TME also features a complex recruitment network of extrinsic signaling pathways. For example, discoidin domain receptor 1 (DDR1) induces CXCL5 via the protein kinase C theta (PKCθ)/spleen tyrosine kinase (SYK)/nuclear factor kappa-B (NF-κB) cascade upon collagen contact (27). Similarly, IL-17 signaling stimulates a broad repertoire of chemoattractants (e.g., CXCL1/3/5 and CSF3) and TNF-α (28). Crucially, both the induction of TNF-α and the inhibition of the KRAS/MEK pathway converge on NF-κB activation to further drive CXCL5 secretion by tumor cells (16).

**Figure 1:**
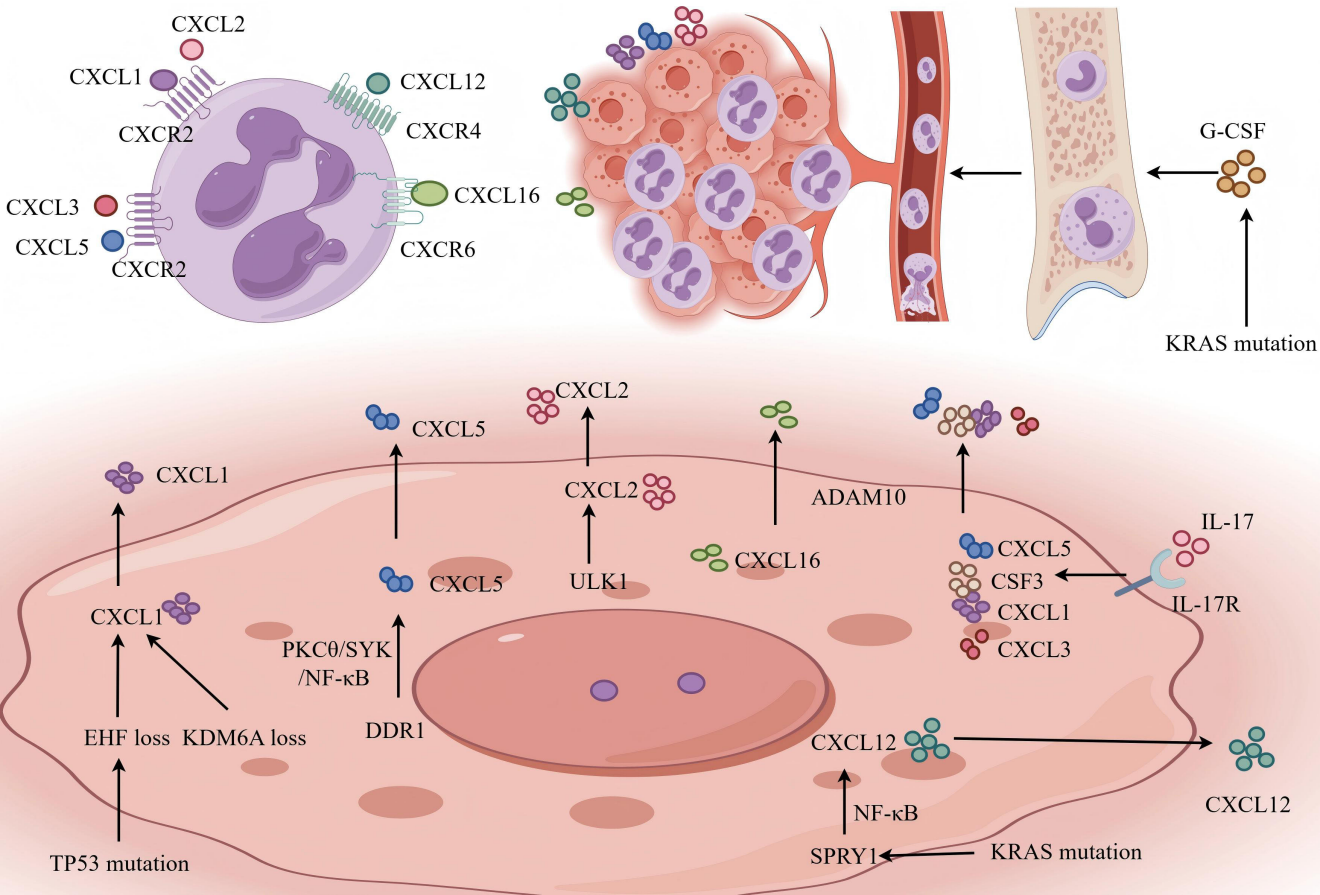
Recruitment mechanisms of neutrophils in PDAC. Constitutive KRAS mutations drive systemic myelopoiesis via G-CSF, while various genetic alterations (e.g., TP53 mutation, KDM6A loss) and extrinsic signals (e.g., IL-17, DDR1) induce the secretion of CXC chemokines (CXCL1/2/5/8) and other ligands (CXCL12, CXCL16). These ligands engage their respective receptors (CXCR2, CXCR4, CXCR6) on circulating neutrophils to facilitate their infiltration. Key regulatory pathways include the NF-κB and PKCθ/SYK pathways. PDAC (pancreatic ductal adenocarcinoma); G-CSF (granulocyte colony-stimulating factor); IL-17 (interleukin-17); DDR1 (discoidin domain receptor 1); CXCL (C-X-C motif chemokine ligand); CXCR (C-X-C motif chemokine receptor); NF-κB (nuclear factor kappa-B); PKCθ (protein kinase C theta); SYK (spleen tyrosine kinase).

Complementing the CXCR2 axis, alternative pathways such as the CXCL12-CXCR4 and CXCL16-CXCR6 axes also facilitate neutrophil influx. For instance, high expression of SPRY1 in KRAS-mutant tumors activates NF-κB to produce CXCL12, while ADAM10-mediated cleavage of membrane-bound CXCL16 releases its soluble form to attract CXCR6+ neutrophil subsets (29, 30). Collectively, PDAC cells exploit a multifaceted network to maintain high ligand concentrations that ensure the continuous recruitment and functional remodeling of TANs (**Figure 1**).

### 2.2 Heterogeneity and functional plasticity of TAN phenotypes

Historically, the functional landscape of TANs was interpreted through a simplistic binary model, categorizing them into anti-tumorigenic (N1) and pro-tumorigenic (N2) phenotypes based on the differential expression of markers such as ICAM-1, CD95, and CXCR2 (CD182) (31). While this framework provided an initial foundation for functional analysis, the advent of single-cell RNA sequencing (scRNA-seq) has revolutionized our understanding, unveiling an intricate degree of TAN heterogeneity within the PDAC microenvironment (11, 32).

Current scRNA-seq evidence delineates multiple functionally distinct neutrophil subpopulations. For instance, four primary subsets have been identified: a terminally differentiated pro-tumorigenic subset (TAN-1) characterized by heightened glycolytic activity; an inflammatory subset (TAN-2); a migratory transitional subset (TAN-3) representing recent tumor infiltrates; and an interferon-stimulated gene (ISG)-high subset (TAN-4) (11). Another study involving 16 PDAC samples further delineated seven neutrophil subsets, identifying Oxidized low-density lipoprotein receptor 1 (OLR1)+ neutrophils and macrophage migration inhibitory factor (MIF)+ neutrophils as tumor-specific TANs that exhibit a prototypical pro-tumorigenic phenotype (32). Additionally, the capacity for neutrophil extracellular trap (NET) formation has emerged as a critical functional axis, with neutrophils categorized into multiple NET-positive and NET-negative clusters (33). Collectively, these findings suggest that TANs do not exist in a rigid N1/N2 dichotomy but rather occupy a continuous spectrum of functional phenotypes. However, technical challenges such as short neutrophil lifespan and low transcriptional activity may limit the full capture of TAN diversity (34).

The functional state of TANs is characterized by dynamic evolution rather than static commitment. Recent studies have unveiled a “convergent reprogramming” paradigm in pancreatic cancer. Regardless of their initial entry state, infiltrating neutrophils eventually converge toward a terminally differentiated dcTRAIL-R1^+^ (T3) state (35). Predominantly localized within the tumor core, these T3 cells exhibit an extended lifespan and specialize in promoting angiogenesis within hypoxic, glycolytic niches, thereby facilitating tumor oxygenation and nutrient acquisition (35).

### 2.3 Polarization of neutrophils

The polarization of neutrophils within the TME is precisely regulated by a myriad of signaling pathways. Transforming growth factor-beta (TGF-β) is a pivotal factor driving neutrophil polarization toward a pro-tumorigenic N2 phenotype (12). conversely, the blockade of TGF-β signaling can induce a transition toward an anti-tumorigenic N1 phenotype. In contrast, interferons (IFNs) are capable of inducing a shift from pro-tumorigenic to anti-tumorigenic phenotypes in both murine and human neutrophils (13). Those studies suggest that TGF-β and Type I IFNs constitute an antagonistic signaling axis governing polarization.

Beyond cytokines, metabolic reprogramming plays a crucial role in the polarization process. Hypoxia-inducible factor-1α (HIF-1α) and the transcription factor BHLHE40 drive the differentiation of neutrophils into pro-tumorigenic subtypes by inducing glycolytic metabolic reprogramming (11, 36). These cells are capable of suppressing the expression of IFN-γ and TNF-α, as well as the proliferative capacity of CD8^+^ T cells (36). Notably, certain pharmacological interventions can reverse this process. For instance, in pancreatic cancer models, melatonin treatment induces a shift toward an N1-like anti-tumorigenic phenotype, characterized by enhanced fatty acid oxidation (FAO) and reactive oxygen species (ROS)-mediated tumor-killing capabilities (37).

These findings demonstrate that neutrophil polarization is not an irreversible terminal state but rather a continuous spectrum dynamically regulated by microenvironmental signals and metabolic status, providing a theoretical foundation for therapeutic reprogramming.

## 3 Anti-tumorigenic functions of TANs

Early studies by Clark and Klebanoff (1975) and Mantovani et al. (1983) first demonstrated neutrophil-mediated tumor cytotoxicity via the myeloperoxidase (MPO) system and antibody-dependent cellular cytotoxicity (ADCC) (38–40). However, the functional duality of neutrophils was only systematically conceptualized after Fridlender et al. proposed the N1/N2 polarization model (12).

At the mechanistic level, the maintenance of the N1 phenotype is closely linked to metabolic reprogramming. Neutrophils utilize fatty acid oxidation (FAO) for energy, driving the NADPH oxidase complex to generate high levels of reactive oxygen species (ROS) and hydrogen peroxide, which mediate direct cytotoxicity (12). Furthermore, arginase-1 (ARG1) released from activated or necrotic neutrophils can trigger the endoplasmic reticulum (ER) stress pathway in cancer cells, thereby inducing apoptosis (41).

Notably, the findings of Granot et al. in a breast cancer model further broadened the dimension of neutrophil anti-tumor function, extending it from the local microenvironment to a systemic level (42). The primary tumor can remotely modulate circulating neutrophils, transforming them into “tumor-entrained neutrophils (TENs)” with anti-metastatic potential. This process is primarily mediated by tumor-derived CCL2, which activates the NADPH oxidase complex in TENs to produce ROS, thereby selectively eliminating metastatic cells in distant organs, such as the lungs (42). In contrast to breast cancer, PDAC-associated neutrophils predominantly exhibit pro-tumorigenic phenotypes characterized by immunosuppressive functions and limited ROS production (43). Whether PDAC can induce anti-tumorigenic neutrophil training similar to breast cancer remains unexplored.

## 4 Pro-tumorigenic functions of TANs

### 4.1 Neutrophil extracellular traps: key pro-tumorigenic effectors

**Figure 2:**
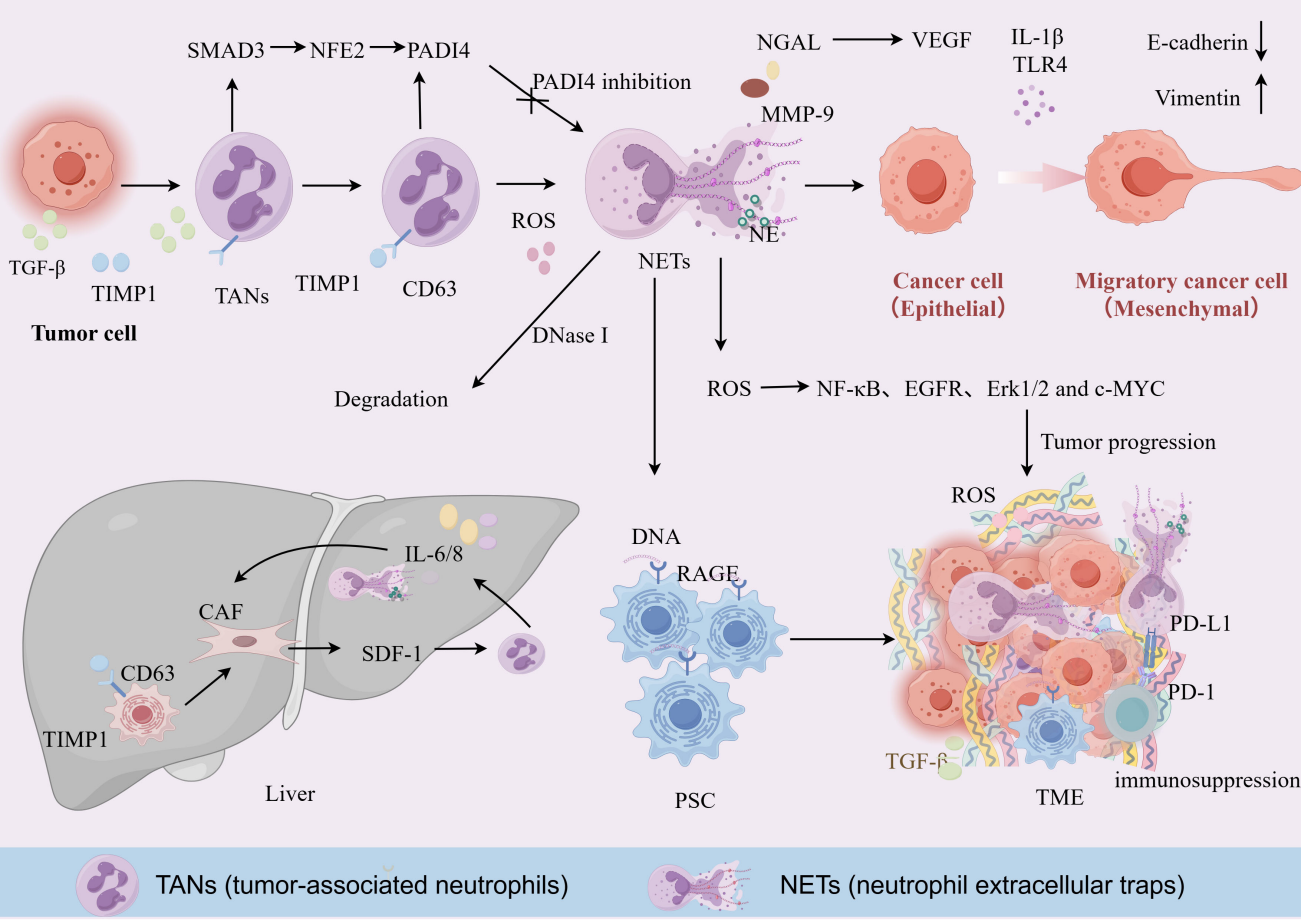
An overview of the mechanisms by which TANs and NETs promote tumor progression and metastasis. In the primary TME, TANs release proteases like NE and MMP-9 (stabilized by NGAL) to facilitate EMT and angiogenesis. Reactive oxygen species (ROS) and cytokines drive NETosis via the PAD4 pathway, which can be inhibited by DNase I or PAD4 inhibitors. In the liver, primary tumor-derived TIMP1 activates hepatic stellate cells (HSCs) via CD63 to recruit neutrophils, creating a pre-metastatic niche. NETs release extracellular DNA that binds to the RAGE receptor on PSCs, stimulating their proliferation and the massive secretion of extracellular matrix promoting desmoplasia. This process hinders T-cell contact with tumor cells and induces T-cell exhaustion by upregulating PD-L1 expression. TANs (tumor-associated neutrophils); NETs (neutrophil extracellular traps); TME (tumor microenvironment); NE (neutrophil elastase); MMP-9 (matrix metalloproteinase 9); NGAL (neutrophil gelatinase-associated lipocalin); EMT (epithelial-mesenchymal transition); ROS (reactive oxygen species); PAD4 (peptidylarginine deiminase 4); TIMP1 (tissue inhibitor of metalloproteinases 1); HSCs (hepatic stellate cells); PSCs (pancreatic stellate cells); PD-L1 (programmed death-ligand 1).

NETs are web-like structures composed of decondensed chromatin DNA associated with granular proteins, including histones, myeloperoxidase (MPO), and neutrophil elastase (NE) (44). The formation of NETs, a process termed NETosis, represents a specialized form of regulated cell death, and also serves as a potent pro-tumorigenic mechanism (44, 45). PAD4 facilitates NETs by catalyzing histone citrullination, a key step that leads to chromatin decondensation, thereby enabling the extracellular release of NETs (46). Concurrently, NE translocates from cytoplasmic granules to the nucleus, where it acts synergistically with PAD4 to further facilitate chromatin decondensation and the eventual extrusion of NETs (47, 48). The critical role of this enzyme is underscored by the fact that PAD4 deficiency completely abrogates the formation of functional NETs (46). Furthermore, tissue inhibitor of metalloproteinases 1 (TIMP1) induces PAD4-dependent histone citrullination by binding to the CD63 receptor on neutrophils and activating the ERK/MAPK signaling axis (49). The receptor for advanced glycation end products (RAGE) enhances the formation of neutrophil extracellular traps (50). IL-17 plays a pivotal role in orchestrating neutrophil recruitment and triggering the formation of NETs, which effectively sequester cytotoxic CD8^+^ T cells, preventing their infiltration into the tumor core (51). NETs promote the malignant progression of PDAC through multifaceted interactions. Within the tumor epithelium, NETs drive the epithelial-mesenchymal transition (EMT), characterized by E-cadherin loss and Vimentin upregulation, thereby bolstering cellular invasiveness (52–54). Additionally, NET-associated IL-1β can activate the EGFR/ERK cascade in cancer cells, further augmenting their migratory potential (55). Beyond direct effects on tumor cells, NETs are master regulators of the desmoplastic stroma. Extracellular DNA from NETs binds to RAGE on pancreatic stellate cells (PSCs), stimulating their proliferation and excessive matrix deposition (56). This resulting fibrotic barrier not only physical restricts the infiltration of therapeutic agents and T cells but also fosters a protective niche for tumor growth (**Figure 2**). Notably, in obesity-associated pancreatic cancer models, NET formation is a key factor driving the progression of pancreatic intraepithelial neoplasia (PanIN) (57).

In the context of metastasis, NETs play a decisive role in establishing the pre-metastatic niche, particularly in the liver. PDAC patients exhibit elevated NET levels in hepatic tissues, which correlate positively with metastatic risk (58). Mechanistically, tumor-derived TGF-β activates the SMAD3 pathway in neutrophils to upregulate the transcription factor NFE2, thereby triggering localized PAD4-dependent NET release at the tumor invasive front. Simultaneously, TIMP1 secreted by primary lesions activates hepatic stellate cells (HSCs) via CD63, inducing the secretion of SDF-1 to recruit additional neutrophils to the liver (**Figure 2**) (59). Once established, NETs promote HSC transdifferentiation into cancer-associated fibroblasts (CAFs) and enhance vascular permeability by stimulating the release of pro-inflammatory cytokines (IL-6, IL-8, and TNF-α) (59). This transformed microenvironment effectively “captures” circulating tumor cells, facilitating their colonization and the subsequent formation of micrometastases. In the infectious microenvironment, Neutrophil Extracellular Traps (NETs) facilitate metastasis by physically sequestering circulating tumor cells (CTCs) and subsequently potentiating their migratory capacity (60).

Finally, a synergistic feedback loop between NET formation and reactive oxygen species (ROS) further amplifies the pro-tumorigenic milieu. While ROS are essential triggers for NETs (47, 61), the resulting NETs facilitate further ROS accumulation within the TME (27, 62). This interplay is highlighted by the fact that MPO deficiency or pharmacological inhibition (e.g., hydroxychloroquine) concurrently suppresses both ROS production and NET release, significantly reducing the intratumoral oxidative burden (63). Within the TME, ROS exert a dualistic role: at moderate concentrations, they activate oncogenic pathways (e.g., NF-κB, c-MYC, and EGFR) to drive proliferation, EMT, and matrix remodeling. However, excessive ROS levels can transition into a cytotoxic state, leading to tumor cell death (43).

Given the central role of NETs, therapeutic strategies targeting PAD4 or deploying DNase I are under active investigation, with particular promise in perioperative settings to prevent recurrence.

### 4.2 TAN-mediated malignant phenotypes in pancreatic cancer

Upon stimulation, neutrophils release a diverse array of antimicrobial enzymes from their cytoplasmic granules into TME (64, 65). These cells characterize four distinct types of granules, each mobilized in a hierarchical manner. Primary (azurophilic) granules sequester potent bactericidal enzymes, including myeloperoxidase (MPO), various hydrolases, cathepsin G (CG), neutrophil elastase (NE), and defensins. Secondary (specific) granules contain lactoferrin along with neutrophil gelatinase-associated lipocalin (NGAL) (64, 65). Tertiary (gelatinase) granules are enriched with matrix metalloproteinase-9 (MMP-9), which is primarily associated with the remodeling of the extracellular matrix (ECM). Finally, secretory vesicles harbor an assortment of pathogen-recognition and complement receptors (64). Beyond bactericidal properties, defensins function as potent chemokines that recruit T cells and monocytes, thereby bridging innate and adaptive immunity. Furthermore, secretory vesicles serve as reservoirs for membrane-associated receptors, harboring molecules such as CD11b/CD18 (Mac-1), fMLP receptors, CD14 (LPS receptor), and CD16. The mobilization and release of these secretory vesicles prompt the cell-surface expression of Mac-1, which mediates firm adhesion to ICAM-1 on endothelial cells (66). CG and NE can deactivate TNFα and cytokine receptors (67, 68). Collectively, these granule-derived immunomodulators are instrumental in regulating immune signaling pathways and mediating non-specific host defense.

Beyond the pro-tumorigenic functions of NETs, the controlled release of diverse proteases during neutrophil degranulation, including NE and MMP-9, serves as a primary engine for TME remodeling (69). These proteases dismantle the ECM, promoting dormant tumor cells proliferation and developing into metastases (70).

NE, the most abundant serine protease in neutrophil granules, functions as a multi-modal driver of malignancy (71–73). NE facilitates epithelial-mesenchymal transition (EMT) by directly cleaving E-cadherin, thereby compromising intercellular junctions and enhancing cellular motility (71, 74). Simultaneously, NE induces tumor progression and migration by the release of TGF-α, PDGF and VEGF (75). At the invasive front, NE degrades core basement membrane components, including laminin and fibronectin, to pave physical pathways for tumor dissemination (76). Furthermore, the resulting ECM degradation products can function as damage-associated molecular patterns (DAMPs), which recruit and activate additional myeloid cells, thereby exacerbating the pro-inflammatory and immunosuppressive milieu (77).

MMP-9 exerts its pro-angiogenic effects primarily through the formation of a stable complex with NGAL (78, 79). This MMP-9/NGAL complex facilitates tumor vascularization by mobilizing matrix-bound vascular endothelial growth factor (VEGF) (80). The functional significance of this axis is evidenced by murine PDAC models, where MMP-9 deficiency leads to a > 50% reduction in microvessel density and a significant attenuation of tumor growth (80). Intriguingly, the NGAL is markedly upregulated as early as the pancreatic intraepithelial neoplasia (PanIN) stage, with expression levels intensifying as the disease advances, underscoring its pivotal role in early malignant transformation (81).

Beyond enzymatic degradation, TANs facilitate metastasis through cytokine secretion and physical interactions. TANs further orchestrate the metastatic cascade through the secretion of pleiotropic cytokines, such as TNF-α and TGF-β, which collectively promote immune evasion and EMT (78). Recent evidence suggests that TANs can form heterotypic aggregates with circulating tumor cells (CTCs). Within these clusters, extracellular vesicle-mediated communication regulates granule mobilization, synergistically enhancing metastatic seeding (82).

Under the systemic influence of PDAC-derived G-CSF, stress-induced myelopoiesis generates an expansion of low-density neutrophils (LDNs) in the peripheral blood (83). While these immature populations exhibit diminished cytotoxicity, they possess a robust secretome that further fortifies the immunosuppressive TME. Additionally, research has revealed that the CXCR2 signaling pathway orchestrates the recruitment of Ly6G^+^ myeloid cells to establish pre-metastatic niches (17). In parallel, mesothelin-induced S100A9 facilitates pulmonary neutrophil infiltration and stimulates localized NET formation (84). Moreover, neutrophil-derived C-C Motif Chemokine Ligand 5 (CCL5) engages the C-C Motif Chemokine Receptor 5 (CCR5) on tumor cells, activating downstream PI3K/AKT and MAPK cascades to upregulate MMP expression, thereby collectively driving the metastatic progression of pancreatic cancer (85).

## 5 Synergistic network interactions between TANs and microenvironmental cells

### 5.1 CAFs

The cross-talk between CAFs and TANs establishes a self-reinforcing pro-tumorigenic milieu. CAFs secrete IL-8, a potent neutrophil activator. Once activated, neutrophils reciprocate by secreting substantial IL-8, forming a positive feedback loop that drives malignant progression (86). The drug pirfenidone (PFD) can disrupt this loop by inhibiting IL-8 secretion in CAF-activated neutrophils. Beyond IL-8 (87), TANs are a major source of TGF-β, which serves as a dual regulator (88). It orchestrates CAF activation and stromal desmoplasia while simultaneously inducing an immunosuppressive phenotype in myeloid cells and impairing T-cell effector functions (89, 90).

Furthermore, IL-1 signaling via the IL1RAP receptor on CAFs triggers the secretion of diverse chemoattractants to recruit neutrophils. These recruited TANs can secrete tumor necrosis factor (TNF) through the CXCR2-MAP3K8-TNF axis. Specifically, the transmembrane form of TNF (tmTNF) binds to TNFR2 on tumor cells or CAFs, stimulating the production of CXCL1. This creates a continuous recruitment cascade that stabilizes the pro-tumorigenic niche (86). Interestingly, this paradigm is not static. Research by Zhou et al. demonstrates that oncolytic herpes simplex virus (oHSV) infection can reprogram CAFs. These reprogrammed CAFs recruit and activate neutrophils toward a phenotype with elevated TNF-α secretion, effectively reshaping the immune microenvironment toward a more inflammatory, anti-tumor state (91).

### 5.2 Tumor-associated macrophages

TANs also synergize with TAMs to construct specialized immunosuppressive hubs. Single-cell RNA-seq analysis revealed significant chemokine-mediated crosstalk between neutrophils and macrophages. Quantitative assessment demonstrated that the abundance of ligand-receptor pairs between these two cell types was substantially higher than that observed for other immune cell pairings (11). Specifically, macrophages orchestrate neutrophil infiltration through the CCL-CCR1 and CXCL-CXCR1/2 axes, while neutrophils, in turn, can recruit macrophages via the CCL3L3-CCR1 signaling pathway (11). In addition, VSIG4, an emerging B7-family immune checkpoint expressed on macrophages, correlates positively with the infiltration of both TAMs and TANs (8). Mechanistically, TAMs secrete osteopontin (SPP1), which directs neutrophil migration by engaging integrin receptors (e.g., CD44), on the neutrophil surface. This interaction leads to the spatial co-localization of these populations into myeloid cell aggregation zones. Multiplex immunofluorescence reveals a profound T-cell exclusion phenomenon within these zones, where CD8^+^ T-cell infiltration is markedly restricted (8).

### 5.3 NK cells

The interaction between TANs and NK cells is characterized by profound context-dependency and bidirectional regulation. Initially, NK cells can exert an anti-tumor effect by secreting IFN-γ, which inhibits VEGF-A expression in neutrophils and prevents their polarization toward a pro-angiogenic N2 phenotype (92). However, TANs have been shown to reshape NK cell functionality through suppressive mechanisms. In immunocompetent settings, while neutrophils may retain residual tumoricidal activity, the reactive oxygen species (ROS) they generate has been observed to impair NK cell cytotoxicity. By impairing the more potent cytotoxic capacity of NK cells, the net biological outcome may shift toward a pro-metastatic state (93, 94). Interestingly, in the absence of NK cells, the direct cytotoxicity of neutrophils can become the dominant force, leading to the paradoxical inhibition of metastasis (93).

Beyond metabolic interference, TANs have been reported to interfere with NK cell immunosurveillance via the Programmed cell Death-1 (PD-1)/Programmed Death-Ligand 1 (PD-L1) signaling axis (95). Driven by tumor-derived G-CSF, TANs activate the STAT3 pathway to upregulate PD-L1 expression. Simultaneously, microenvironmental IL-18 induces PD-1 expression on NK cells (95). This checkpoint activation exerts a multifaceted inhibitory effect on NK cells. It not only diminishes lytic activity by desensitizing NKp46 and NKG2D activating receptors but also orchestrates the downregulation of CCR1, thereby hindering the migratory and infiltrative capacity of NK cells (95). By impairing the more potent cytotoxic capacity of NK cells, the net biological outcome may shift toward a pro-metastatic state (96).

### 5.4 Effector T cells and Tregs

In addition to potentiating tumor development, TANs function as critical mediators that underpin the immunosuppressive microenvironment. TANs drive immunosuppression, establishing a formidable barrier against T-cell-mediated immunity through the expression of PD-L1, Nectin2, and the secretion of CCL5 (85).

In PDAC, a specific p2rx1^-^ TAN subpopulation, characterized by high PD-L1 expression and enhanced mitochondrial metabolism, is enriched in liver metastases. This subset is characterized by high PD-L1 expression and enhanced mitochondrial metabolic reprogramming, potentially supporting its sustained suppressive function through metabolic adaptation. Mechanistically, PD-L1-positive TANs directly inhibit the proliferation and cytotoxicity of T cells via PD-1 binding—an effect that can be selectively reversed by anti-PD-1 neutralizing antibodies. This TAN-mediated suppression is not idiosyncratic to PDAC but appears to be a pan-cancer phenomenon. For instance, in breast cancer, the chemokine CCL20 induces PD-L1 expression in TANs, which correlates with diminished T-cell immunity and poor disease-free survival. Similar paradigms have been observed in hepatocellular and gastric cancers, underscoring the PD-L1/PD-1 axis as a conserved driver of TAN-mediated immune evasion (93, 94).

Furthermore, the hostile PDAC microenvironment defined by hypoxia and nutrient deprivation triggers endoplasmic reticulum stress (ERS) (97, 98). Under ERS signaling, TANs polarize toward an immunosuppressive phenotype characterized by the upregulation of CCL5 and Nectin2 (85). Nectin2 is thought to engage the inhibitory receptors CD112R (PVRIG) or TIGIT on T cells, delivering signals that block T-cell receptor (TCR) activation and attenuate IFN-γ production. Concurrently, TAN-derived CCL5 facilitates the recruitment of Tregs into the tumor core. Once infiltrated, these Tregs secrete inhibitory cytokines like TGF-β and IL-10, which not only suppress effector T cells but also reinforce the N2 polarization of TANs (85).

## 6 The critical role of TANs in treatment resistance and tumor recurrence

Beyond their fundamental role in immunosuppression, TANs are central orchestrators of therapeutic resistance in PDAC, contributing to the failure of chemotherapy and immunotherapy as well as driving postoperative recurrence. A significant treatment paradox exists in PDAC.

### 6.1 Chemotherapy resistance

Chemotherapy induces metastatic cancer cells to secrete CXCL1 and CXCL2, which in turn promote the directional recruitment of neutrophils to the liver. These neutrophils exhibit high expression of Gas6, which activates downstream signaling by engaging the AXL receptor on cancer cells, thereby driving the post-chemotherapy proliferation of metastatic tumor cells (99). Furthermore, gemcitabine plus nab-paclitaxel (GnP) treatment has been shown to orchestrate neutrophil recruitment to the TME, subsequently driving therapeutic resistance in PDAC murine models. This process is mediated by the upregulation of GPRC5A or the direct induction of IL-8 (CXCL8) secretion by gemcitabine, which activates the CXCR1/2 signaling axis on neutrophils to promote NETosis (100, 101). These NETs serve as a critical barrier that contributes to the recalcitrance of PDAC to cytotoxic therapies. The resulting cell-free DNA (cfDNA) from NETs provides a scaffold that facilitates cancer cell migration and metabolic survival (101). Experimental evidence suggests that degrading cfDNA with DNase I, inhibiting PAD4, or utilizing CXCR1/2 inhibitors (e.g., Navarixin) can significantly sensitize PDAC to chemotherapy (100).

The complexity of resistance is further amplified by the interplay between neutrophils and the stroma. Neutrophil-derived TNF (and its expression in PMN-MDSCs) induces the polarization of CAFs toward an inflammatory phenotype. Blocking this loop with TNFR2 inhibitors has been shown to enhance chemotherapy sensitivity (86). Additionally, in chemoresistant tumors, the upregulation of ZEB1 promotes the secretion of SPP1 (Osteopontin), which recruits neutrophils via CD44 and drives their polarization toward an immunosuppressive state, ultimately triggering CD8+ T-cell exhaustion (102). In the context of KRAS mutation, the RAS/MEK/ETS2 axis induces robust G-CSF secretion, mobilizing neutrophils that secrete Bv8 (Prokineticin 2, PK2) (103). Notably, when tumors become refractory to classic anti-VEGF therapies (e.g., bevacizumab), they often switch to the PK2/Bv8 pathway as an alternative angiogenic driver, sustaining vascularization and disease progression.

### 6.2 Immunotherapy resistance

Despite the high expression of PD-L1 on TANs suggesting potential for anti-PD-1 sensitization, clinical outcomes remain suboptimal. Perioperative GVAX combined with PD-1 blockade and CD137 agonism (triple therapy) for pancreatic cancer increased intratumoral activated cytotoxic T-cell infiltration and showed potential DFS/OS benefit signals. However, no statistically significant survival advantage was achieved, possibly due to compensatory neutrophil infiltration (104). Data from trials combining GVAX with Nivolumab indicate that a high density of pre-treatment TANs serves as a robust indicator of poor prognosis (18). This suggests that TANs possess redundant immunosuppressive mechanisms beyond the PD-1/PD-L1 axis. Anti-PD-1 treatment may induce CD8^+^TNFRSF9^+^ T cells to act on neutrophil receptors via the expression of ligands such as IFN-γ, inducing neutrophil degranulation. This process extensively remodels the extracellular matrix (ECM), creating a dense biophysical barrier that physically excludes subsequent T-cell infiltration. It serves as a critical driver of acquired resistance to PD-1 inhibitors in PDAC (18). In addition, TANs exhibit high expression of CXCR2, which correlates with an accumulation of LAG3+ exhausted T-cells, suggesting that TANs may facilitate therapeutic resistance by inducing T-cell exhaustion (18).

While CD40 agonists are designed to orchestrate systemic immune activation, their clinical efficacy is markedly constrained in tumors harboring the Trp53R172H mutation. This specific mutation triggers a massive infiltration of neutrophils, which construct a robust immunosuppressive barrier. Even upon systemic T-cell activation by CD40 agonists, these intratumoral neutrophils continue to suppress T-cell proliferation and effector function, as well as the maturation of antigen-presenting cells (APCs). Consequently, this prevents T cells from effectively infiltrating the tumor parenchyma or exerting potent cytotoxic activity (24).

In addition, NETs are critical drivers of immune resistance in PDAC. By effectively sequestering cytotoxic CD8^+^ T cells within their DNA scaffolds, NETs prevent lymphocytes from reaching the tumor parenchyma (51). Furthermore, NETs act as immunosuppressive niches by concentrating PD-L1, which directly promotes T-cell exhaustion (105). Consequently, targeting the IL-17/NETs axis, either through Pad4 inhibition, DNase I treatment, or IL-17 blockade, offers a promising strategy to circumvent resistance to ICB (e.g., anti-PD-1) and restore anti-tumor immunity (51, 105).

TANs form a critical barrier in PDAC by driving phenotypic polarization and building physical niches. Consequently, therapeutic interventions that block neutrophil recruitment or reprogram their function could effectively mitigate resistance and recurrence.

## 7 Therapeutic strategies targeting neutrophils

Given the profound regulatory role of neutrophils in the PDAC TME, targeting neutrophils and their associated pathways has become a forefront area of research. These strategies can be broadly categorized into three main classes: inhibiting neutrophil generation and recruitment, modulating their functional polarization and activity, and blocking their immunosuppressive effects (**Table 1**).

**Table 1:** Therapeutic strategies targeting neutrophils in the tumor microenvironment for pancreatic cancer.

Therapeutic strategy	Target/pathway	Representative agent	Research phase/NCT ID	Mechanism of action	References
Inhibition of Development & Recruitment	FES (ALK/ROS1)	Lorlatinib	Preclinical	Suppresses bone marrow myelopoiesis and impairs and recruitment to the tumor site.	(102)
	CXCR1/2	CXCR1/2 antagonist navarixin	Preclinical clinical trial	Blocks the chemokine axis to inhibit neutrophil migration into the TME.	(95)
	CXCR1/2	CXCR1/2 antagonist navarixin	Phase II clinical trial (Terminated)	Targeted recruitment; trial terminated due to lack of efficacy and suboptimal target engagement.	(104)
	CXCR2	CXCR2 pepducin (X1/2pal-i3)	Preclinical	Small-molecule inhibitor that suppresses metastatic spread when combined with gemcitabine.	(17)
	IL1RAP	Nadunolimab	Phase II clinical trial	Blocks CAF-mediated recruitment and improves outcomes in high-IL1RAP expressing patients.	(106)
	IL-8	IL-8 Blocker	Clinical trial (NCT02451982)	Combined with GVAX vaccine and CD137 agonist to optimize T-cell responses.	(99)
	TNFR2	Etanercept	Preclinical	Disrupts the self-amplifying circuit between PMN-MDSCs and CAFs.	(81)
Functional Reprogramming (N2 → N1)	TGF-βR Inhibitor	Nanoparticles	Preclinical	Abrogates N2 polarization and favors an anti-tumor N1 phenotype.	(107)
	TGF-β	Galunisertib	Clinical	Combined with Durvalumab; showed limited activity in heavily pretreated metastatic patients.	(108)
	PD-L1/TGF-βRII	SHR-1701	Clinical trial (NCT04624217)	Bispecific fusion protein that neutralizes both PD-L1 and TGF-β signaling.	(109)
	Metabolism	Melatonin	Preclinical	Induces N1-like phenotype by enhancing fatty acid oxidation and ROS-mediated killing.	(37)
Disruption of Suppression & NETs	PAD4	PAD4 Inhibitor	Preclinical	Prevents histone citrullination to block the formation of NETs (NETosis).	(110)
	NET Structure	DNase I	Preclinical	Enzymatically degrades the DNA scaffold of NETs to restore T-cell functionality.	(101)
Reversal of Myeloid-Mediated Immune Suppression	CCR5	Maraviroc	Preclinical	Blocks the CCL5/CCR5 axis to diminish Treg infiltration and facilitate T-cell priming.	(91)
	ARG1	INCB001158	Phase I/II clinical trial (NCT02903914)	Inhibits arginase-1 to restore CD8^+^ T-cell metabolic activity and function.	(111)

### 7.1 Inhibiting neutrophil development and recruitment

The ALK/ROS1 inhibitor lorlatinib has been identified as a potent inhibitor of the non-receptor tyrosine kinase FES (106). In PDAC models, lorlatinib markedly attenuates tumor progression through a multi-pronged mechanism. It suppresses neutrophil myelopoiesis in the bone marrow, impairs their subsequent recruitment to the tumor site, and neutralizes the pro-proliferative signaling exerted by TANs (106). Notably, when integrated with anti-PD-1 immunotherapy, lorlatinib significantly augments the infiltration of primed CD8^+^ T cells, eliciting a robust synergistic anti-tumor response (106).

Given that the majority of TANs highly express CXCR2, targeting this receptor represents a strategic approach to disrupting neutrophil-mediated immunosuppression. In KPC mice, treatment with CXCR2 pepducin inhibitors or small-molecule inhibitors (e.g., AZ13381758) significantly prolonged survival. These effects were particularly pronounced when combined with gemcitabine, which led to extended lifespans and the complete suppression of metastatic spread (17). Furthermore, the combination of CXCR2 blockade and anti-PD-1 therapy has demonstrated superior survival outcomes in preclinical settings (17, 107). Despite its safety, the combination of Navarixin and Pembrolizumab failed to show clinical efficacy in multiple solid tumors and was terminated for futility (NCT03473925), casting doubt on whether CXCR2 inhibition alone can sufficiently neutralize MDSC activity to enhance immunotherapy (112).

Beyond direct CXCR2 inhibition, alternative strategies involve modulating the upstream drivers of neutrophil recruitment. A clinical study utilizing the GVAX vaccine incorporated a combination of an IL-8 blocker and a CD137 agonist (NCT02451982) to optimize T-cell responses by reprogramming TAN function (104). However, recent evidence suggests that the myeloid compartment possesses significant plasticity. Targeting either CCR2 (regulating macrophages) or CXCR2 (regulating neutrophils) in isolation often triggers compensatory infiltration of the alternative myeloid subset, thereby sustaining the immunosuppressive state (108). Consequently, dual CCR2/CXCR2 blockade has emerged as a superior strategy. Preclinical data confirm that dual inhibitors, when paired with chemotherapy, significantly reduce total myeloid infiltration while increasing the density and activation of intratumoral CD8^+^ T cells (108).

Molecular insights have further identified the EHF transcription factor as a critical regulator whose loss upregulates CXCL1, driving the recruitment of CXCR2^+^ neutrophils and fostering therapeutic resistance (23). The agent nifuroxazide can restore EHF expression and inhibit the JAK1/STAT1 pathway, thereby reducing neutrophil influx and sensitizing PDAC to chemo- and immunotherapy. Similarly, IL-17 promotes tumor progression by both recruiting neutrophils via CXCL1/5 and inducing PAD4-dependent NETosis. Combined anti-IL-17 and anti-PD-1 therapies have shown significant synergy in PDAC models (28). Additionally, IL-1 signaling on CAFs via the IL1RAP receptor facilitates chemokine-mediated neutrophil recruitment (109). The IL1RAP antibody nadunolimab combination with gemcitabine and nab-paclitaxel showed promise with improved clinical outcomes observed in patients with advanced/metastatic pancreatic cancer who harbor high IL1RAP expression in Phase I/II trials (113).

Finally, recent studies highlight a sophisticated feedback loop involving Polymorphonuclear myeloid-derived suppressor cells (PMN-MDSCs), which produce tmTNF via the CXCR2-MAP3K8-TNF axis. This tMTNF binds to TNFR2 on both tumor cells and CAFs, triggering further CXCL1 secretion and creating a self-amplifying circuit for PMN-MDSC recruitment (86). This axis also drives CAFs toward a pro-inflammatory, IL-6-high phenotype. Disrupting this circuit with a TNFR2 inhibitor (e.g., etanercept) offers a dual therapeutic advantage. It reduces PMN-MDSC infiltration and reverts CAF activation, ultimately overcoming T-cell exclusion and chemoresistance in PDAC (86).

### 7.2 Targeting TAN polarization and mediated immunosuppression

Reprogramming TANs from a pro-tumor N2-type to an anti-tumor N1-type is a pivotal strategy for modulating the PDAC immune landscape. Actively steering this polarization can significantly potentiate the efficacy of immunotherapy. Polymorphonuclear neutrophils (PMNs) exert ADCC activity following human/mouse chimeric antibody Nd2 (c-Nd2) stimulation. Granulocyte-colony stimulating factor (G-CSF) potentiates c-Nd2-induced ADCC activity (39). Consequently, the combination of c-Nd2 and G-CSF significantly inhibits pancreatic cancer xenograft growth and demonstrates considerable potential for clinical translation (38). In addition, in a regimen combining irreversible electroporation (IRE) ablation with anti-PD-1 therapy, the localized delivery of TGF-β receptor inhibitor nanoparticles effectively abrogated the N2-polarization of infiltrating neutrophils, instead favoring their conversion to an anti-tumor N1 phenotype (110). This combinatorial approach significantly extended survival in murine models and established robust long-term anti-tumor immune memory.

The central role of TGF-β in maintaining the immunosuppressive TME is further underscored by the use of TGF-β ligand traps (TGF-β-TRAP) (111). These traps remodel the heterogeneous PDAC microenvironment by reprogramming both CAFs and myeloid cells into pro-inflammatory agonists (111). In metastasis-prone models, this therapy paired with anti-PD-1 significantly diminished the metastatic burden, likely by modulating the CCL5/CCR5 axis and shifting CCR5 signaling from an immunosuppressive (via CCL5) to an immune-agonist state (via CCL7/CCL8) (111). Despite these promising preclinical results, the clinical translation of TGF-β inhibitors has faced hurdles. Data for the TGF-β inhibitor galunisertib combined with durvalumab showed manageable safety but limited clinical activity in heavily pretreated metastatic PDAC patients (114). This discrepancy suggests that future efforts must focus on precise patient stratification or optimizing the timing of intervention. Conversely, in the first-line setting, SHR-1701, a bispecific fusion protein targeting both PD-L1 and TGF-βRII combined with chemotherapy, has demonstrated significant anti-tumor activity (NCT04624217) (115). By simultaneously neutralizing the PD-1/PD-L1 axis and TGF-β signaling, this agent effectively reverses TME-mediated suppression with a favorable safety profile.

Despite promising preclinical data, TGFβ pathway inhibitors have shown limited single-agent activity in PDAC patients. Understanding why these therapies fail is critical for developing more effective strategies. A fundamental challenge stems from TGFβ’s dual roles: tumor-suppressive in epithelial cells but tumor-promoting in the microenvironment (116). Approximately 55% of PDAC patients have SMAD4 loss, rendering their tumors insensitive to TGFβ-mediated growth inhibition while retaining microenvironmental effects (117). This creates a therapeutic dilemma. While TGF-β inhibitors block late-stage metastasis and immunosuppression, they may also relieve growth-inhibitory pressure on tumor cells, potentially yielding antagonistic effects that offset clinical benefits. This paradox underscores the complexity of targeting a pathway with opposing roles depending on cellular context and disease stage. Beyond patient heterogeneity, the limited efficacy observed in clinical trials reflects suboptimal patient selection and disease timing. Those immunogenic individuals enriched CD4+ and CD8+ T cell signaling are more likely to respond to immunotherapy (118). Most clinical trials have enrolled heavily pre-treated patients with advanced-stage disease. Late-stage tumors accumulate multiple resistance mechanisms, have depleted T cell repertoires, and show extensive clonal heterogeneity. Therapeutic strategies should prioritize early-line patients with high levels of IP-10 and MIP-1α (119). An additional layer of complexity arises from stromal reprogramming. TGFβ inhibition shifts CAFs from tumor-restraining myCAFs to pro-inflammatory iCAFs, potentially having pro-tumorigenic consequences through enhanced cytokine secretion interleukin 6 (IL-6) (120). Moreover, TGFβ signaling does not operate in isolation but engages in extensive crosstalk with multiple oncogenic pathways including ERK/MAPK, PI3K/AKT, WNT, and HIPPO-YAP (121). Notably, TGFβ-induced EMT and migration often involve non-canonical pathways such as ERK, ROS signaling, and RAC1 activation (121). Consequently, blocking TGFβ receptors alone may prove insufficient, as tumor cells can activate these bypass signaling routes to maintain survival and migratory capacity. This phenomenon of adaptive pathway activation highlights the need for rational combination strategies that simultaneously target TGFβ and its key downstream or parallel effectors to achieve durable therapeutic responses.

Beyond TGF-β, other metabolic and stress-related pathways offer novel targets for TAN reprogramming. Blocking the immune checkpoint molecule (CD112), which is preferentially expressed on a TAN subpopulation driven by BHLHE40, or inhibiting endoplasmic reticulum stress (ERS) signaling, has been shown to alleviate T-cell exhaustion (85).

Targeting NETs via either the enzymatic degradation of their structure with DNase I or the prevention of their biogenesis through PAD4 inhibition can effectively alleviate T-cell suppression (122, 123). TANs also orchestrate the recruitment of Tregs via high expression of CCL5. Consequently, the use of CCL5-neutralizing antibodies or CCR5 antagonists (e.g., maraviroc) can diminish Treg infiltration and facilitate CD8^+^ T-cell function (85). Moreover, N2-type TANs express high levels of arginase-1 (ARG1), which depletes local arginine levels and stunts T-cell metabolic activity (124). Combining ARG1 inhibitors with immune checkpoint inhibitors (ICIs) has shown potential in restoring CD8^+^ T-cell function within the nutrient-depleted PDAC extracellular environment. Clinical evaluation in a Phase I/II trial (NCT02903914) assessing the ARG1 inhibitor INCB001158 both as a monotherapy or in combination with pembrolizumab for metastatic solid tumors shows INCB001158 was generally well-tolerated, but demonstrated limited anti-tumor activity (125).

## 8 Research status, challenges, and prospects

TANs have emerged as central immune regulators in PDAC, characterized by profound functional plasticity and a distinct dual role. On the pro-tumorigenic front, TANs drive metastasis, immune evasion, and therapeutic resistance through multifaceted mechanisms, including NET formation, establishment of the CXCL1-CXCR2-TNF positive feedback loop, and epigenetic reprogramming. Notably, NET-mediated sequestration of effector T cells and TAN-induced ECM remodeling are primary drivers of chemoresistance and acquired resistance to immune checkpoint blockade. Conversely, targeted interventions such as TGF-β blockade can reprogram TANs toward an anti-tumor phenotype, highlighting their therapeutic malleability. In clinical settings, peripheral blood induces particularly the neutrophil-to-lymphocyte ratio (NLR) alongside intratumoral NET density, have emerged as robust biomarkers for prognostic stratification and therapeutic response prediction (6, 126).

Despite compelling preclinical evidence supporting TAN-targeted therapies, clinical translation has encountered significant obstacles (107, 127). Clinical evaluations of both ARG1 and CXCR2 inhibitors have consistently demonstrated manageable safety and tolerability; however, their clinical efficacy remains underwhelming (112, 125). These outcomes underscore the intricate complexity of metabolic and chemokine signaling within the tumor microenvironment (TME). The lack of clear clinical benefit suggests that targeting a single axis such as arginine metabolism or CXCR2-mediated recruitment may trigger compensatory immunosuppressive pathways. Consequently, these findings highlight a critical need for more sophisticated intervention strategies, including the identification of precise patient sub-populations through predictive biomarkers and the development of synergistic combination therapies that address the multi-layered nature of myeloid-mediated immunosuppression. Furthermore, the traditional N1/N2 binary classification is increasingly recognized as an oversimplification. The field currently lacks unified, high-specificity molecular markers to precisely identify and target pro-tumorigenic TAN subpopulations, complicating the design of selective therapeutic strategies.

Future research must address these translational bottlenecks through several key directions. First, leveraging single-cell multi-omics (scRNA-seq, scATAC-seq) and spatial transcriptomics will be essential to delineate high-resolution dynamic landscapes of TAN polarization, enabling the identification of stage-specific intervention windows and targetable subpopulations. Second, the therapeutic paradigm should shift from non-specific neutrophil depletion toward “directed functional reprogramming. Strategies such as blocking Nectin2 (CD112), modulating ERS signaling, or targeting metabolic checkpoints (e.g., BHLHE40, HIF-1α) hold promise for reverting TAN phenotypes while preserving host innate immunity (11, 36, 85). Third, combination regimens integrating novel agents (e.g., CXCR1/2 inhibitors, lorlatinib, nifuroxazide) with immune checkpoint inhibitors and standard chemotherapy require rigorous clinical validation in appropriately stratified patient cohorts. Finally, the integration of dynamic biomarkers including NLR, circulating NET components (e.g., citrullinated histone H3), and imaging-based assessments (e.g., the PORCELAIN AI model) into routine clinical practice will be critical for real-time treatment guidance and outcome prediction.

## 9 Conclusion

In conclusion, while TAN heterogeneity and myeloid compensatory mechanisms present formidable challenges, the convergence of advanced profiling technologies, mechanistic insights, and innovative therapeutic strategies positions TANs as a pivotal, actionable target in PDAC. Transitioning from neutrophil depletion to the targeted reprogramming of these plastic regulators offers a paradigm shift to redefine the PDAC treatment landscape.
